# Validation of a Pre-Coded Food Diary Used among 60–80 Year Old Men: Comparison of Self-Reported Energy Intake with Objectively Recorded Energy Expenditure

**DOI:** 10.1371/journal.pone.0102029

**Published:** 2014-07-14

**Authors:** Tonje H. Stea, Lene F. Andersen, Gøran Paulsen, Ken J. Hetlelid, Hilde Lohne-Seiler, Svanhild Ådnanes, Thomas Bjørnsen, Svein Salvesen, Sveinung Berntsen

**Affiliations:** 1 Department of Public Health, Sport and Nutrition, University of Agder, Kristiansand, Norway; 2 Department of Nutrition, Institute of Basic Medical Sciences, University of Oslo, Oslo, Norway; 3 Department of Physical Performance, Norwegian School of Sport Sciences, Oslo, Norway; Sookmyung Women's University, Republic of Korea

## Abstract

**Objective:**

To validate energy intake (EI) estimated from a pre-coded food diary (PFD) against energy expenditure (EE) measured with a valid physical activity monitor (SenseWear Pro_3_ Armband) and to evaluate whether misreporting was associated with overweight/obesity in a group of elderly men.

**Methods:**

Forty-seven healthy Norwegian men, 60–80 years old, completed the study. As this study was part of a larger intervention study, cross-sectional data were collected at both baseline and post-test. Participants recorded their food intake for four consecutive days using food diaries and wore SenseWear Pro_3_ Armband (SWA) during the same period. Only participants with complete data sets at both baseline and post-test were included in the study.

**Results:**

The group average EI was 17% lower at baseline and 18% lower at post-test compared to measured EE. Mean difference from Bland-Altman plot for EI and EE was −1.5 MJ/day (±1.96 SD: −7.0, 4.0 MJ/day) at baseline and −1.6 MJ/day (−6.6, 3.4 MJ/day) at post-test. The intraclass correlation coefficient (ICC) was 0.30 (95% CI: 0.02, 0.54, p = 0.018) at baseline and 0.34 (0.06, 0.57, p = 0.009) at post-test. Higher values of underreporting was shown among overweight/obese compared to normal weight participants at both baseline and post-test (p≤ 0.001), respectively.

**Conclusions:**

The results indicate that the PFD could be a useful tool for estimating energy intake in normal weight elderly men. On the other hand, the PFD seems to be less suitable for estimating energy intake in overweight/obese elderly men.

## Introduction

In European countries there is a growing elderly population, and it is predicted that the current 15% of the total population aged 65 or more years will increase to more than 25% by 2050 [1]. A similar growth rate of the elderly population is predicted in America and Australia [2], [3]. As this is the fastest growing segment of the population, it becomes more apparent that investments in aging and health, including nutrition is essential. In several studies in older adults a relationship between dietary patterns and dietary quality and obesity-related health outcomes and mortality have been reported [4]–[7]. However, nutrition science is hampered by the fact that there is a questionable precision in most methods for dietary assessments [8]–[10].

A general finding in dietary studies is the tendency to underreport energy intake, and this is found both among children and adolescents [11], [12], adults [13], [14] as well as elderly [15], [16]. In a study by Sharhar et al. [15] among high-functioning community-dwelling elderly, 70–79 years old, it was shown that underreporters had significantly higher body weight than the rest of the participants. A Danish cohort study, examining men at the mean ages of 20, 33, 44, and 49, has also shown that underreporting was more prevalent in obese men than those who were not obese [14].

In several studies energy expenditure (EE) has been estimated by the doubly labelled water (DLW) method to assess the possible disparity between EE and energy intake (EI), where EI is measured with either weighed or estimated methods [15], [17]. The reason for using EE to validate EI is because there are no biochemical biomarkers of EI, so the methods of validation rest on the assumption that EI must be equal to EE when weight is stable [10]. Although the DLW method is clearly the most accurate method for measuring average EE, its use is limited in large groups because of its high cost, both for the labelled water, for the specialised equipment for the analysis and for the trained personnel [18]. Johannsen et al. [19] have reported that SenseWear Pro_3_ Armband (SWA; BodyMedia Inc., Pittsburg, PA, USA) register energy expenditure in healthy adults similar to or even more accurate than other available monitors during 14 days of monitoring. A reasonable level of concordance was demonstrated between SWA and DLW methods, both in the latter mentioned study (ICC = 0.63) and in another study (ICC = 0.46) for measuring daily EE in free-living adults during 10 days of monitoring [20]. Thus, comparison of different methods showed that SWA seemed to be a relatively inexpensive, practical and accurate monitor of EE.

The aim of the present study was to validate energy intake (EI) estimated from a pre-coded food diary (PFD) against energy expenditure (EE) measured with the SWA. Furthermore, to evaluate whether misreporting was associated with overweight/obesity in a group of Norwegian elderly men aged 60–80 years.

## Subjects and Methods

### Ethics Statement

The study has been approved by the Norwegian Regional Committee for Medical Ethics South-East C (2010/1352). This is an independent committee, appointed by the Norwegian Ministry of Education, IRB 00001870. Written informed consent was obtained from all the participants. The trial registration number was ACTRN12614000065695.

### Subjects

Healthy men between 60–80 years old were invited to participate in the study and the participants were recruited in the south of Norway through advertisement in a local newspaper. A total of 200 men showed up at an open information meeting, and those who were healthy, non-smokers, did not use dietary supplements or any kind of medications that was likely to affect the results of the main study were invited to participate (n = 71). Medications to treat high cholesterol, blood pressure, migraine, and mild antidepressants were accepted. To ensure that the subjects were able to participate in the intervention study, a cardiologist at Sørlandet hospital, Kristiansand, conducted a medical screening before entering the study. Exclusion criteria included any overt disease, including COPD, cancer and heart disease. As a result of the health screening, 16 of the invited participants were excluded from the study. In addition, two subjects decided to drop out of the study due to personal circumstances. During the intervention, three more dropped out of the study due to a hip operation, a broken ankle and a biceps rupture, respectively. For analyzes, another three participants were excluded due to incomplete data sets. Thus, 47 participants completed the baseline study and the data sets were used in the analysis described in this report.

### Design

This validation study is part of a larger double-blinded randomized placebo-controlled trial with aim to investigate whether supplementation with the antioxidants vitamin C and vitamin E may enhance adaptations to 12 weeks of strength training in terms of muscle growth and increase maximal strength in elderly men. The present study was initiated by the University of Agder in partnership with Norwegian School of Sports Science and Sørlandet hospital, Kristiansand.

Collection of data for the present study was carried out at two different occasions; in August (baseline) and December 2012 (post-test). The participants were given both written and oral instruction on how to fill out the PFD and how to use the SWA. It was emphasized that the participants should not change eating- and activity patterns during the measurement period. Studies has confirmed that 3–5 days of monitoring is required to reliably estimate habitual physical activity, and 4–7 recording days is required to reliably estimate energy intake using a PDF in adults [11], [21]. During both periods of data collection, the monitoring period was 4 days; the participants recorded their entire food intake for one weekend day and three consecutive weekdays and wore the SWA during the same period. Trained researchers telephoned all participants on the second day of the recording period to answer any questions and correct misunderstandings. Participants also received contact information, in order to ask questions to be answered at any time by the trained researchers.

### Food Diary and photographic booklet

The PFD, using household measures and photographs for portion size estimation, was originally developed for use among Norwegian children and adolescents [22]. The PFD method provides a detailed dietary registration as it included questions about consumption of 277 food items grouped together according to the typical Norwegian meal pattern [23]. Each food group was supplemented with open-ended alternatives. The design of the PFD was similar to a cross-table with food listed on the left and time span across the top. Food amounts were presented in predefined household units (e.g. glasses, pieces or tablespoons) or as portions estimated from photographs. Along with the food diary, each participant received a validated photography-booklet that contained thirteen series of coloured photographs, each with four different portion sizes ranging from small to large [24]. The participants were instructed to register food and beverage intake immediately after each meal throughout the day. The diaries were scanned using the Teleform program, version 6.0 (Datascan, Oslo, Norway). Daily intake of energy was computed using the food database and software system (KBS, 2012), developed at the Department of Nutrition, University of Oslo. The food database is mainly based on the official food composition table [25].

### SenseWear Pro_3_ Armband (SWA)

The SWA is a portable device that monitors physiological parameters, including heat flux, skin temperature, galvanic skin response and skin temperature, and movement (bi-axial accelerometer) [20]. The participants were instructed to wear the SWA in order to register each day during the data collection period, starting from midnight at the first day of registration. They were instructed on how to apply the armband and informed that the armband should be worn at all times except when taking a bath or shower. The SWA was worn on the right arm over the triceps branchii muscle at the midpoint between the acromion and olecranon processes [20] and data were computed in 1-minute intervals. The participant's SWA data were acceptable for analysis if overall wear time was ≥19.2 hours/day during the period of data collection. SWA has been validated in adult populations, and the results showed underestimation of total EE with 4.7% and 12.5%, compared to estimates derived from doubly labelled water [19], [20] and 9% compared to estimates derived from indirect calorimetry [26].

### Weight, height, body mass index and lean mass measurements

Body weight and height were measured by trained project staff at two times during each data collection at baseline and post-test, respectively, and mean weight and height for both times were used for statistical analyses. Weight was measured with subjects in light clothing (shorts and t-shirt), and height was measured to the nearest 0.5 cm, using a measuring tape and body-mass monitor (Seca optima), respectively. Body mass index (BMI) was calculated as weight divided by the square of height (kg/m^2^). Criteria for overweight, and obesity used in the present study were consistent with the definitions set forth by the World Health Organization (WHO) where overweigh  =  BMI 25.0–29.9 kg/m^2^ and obesity  =  BMI ≥30 kg/m^2^ [27]. Fat mass measured by one experienced observer was assessed by dual-energy X-ray absorptiometry (DXA; GE-Lunar Prodigy, Madison, WI, USA), which is currently recognized as a well-established reference method for measuring body composition [28], at both baseline and post-test. Participants were scanned from head to toe in supine position.

### Statistical methods

**Figure 1:**
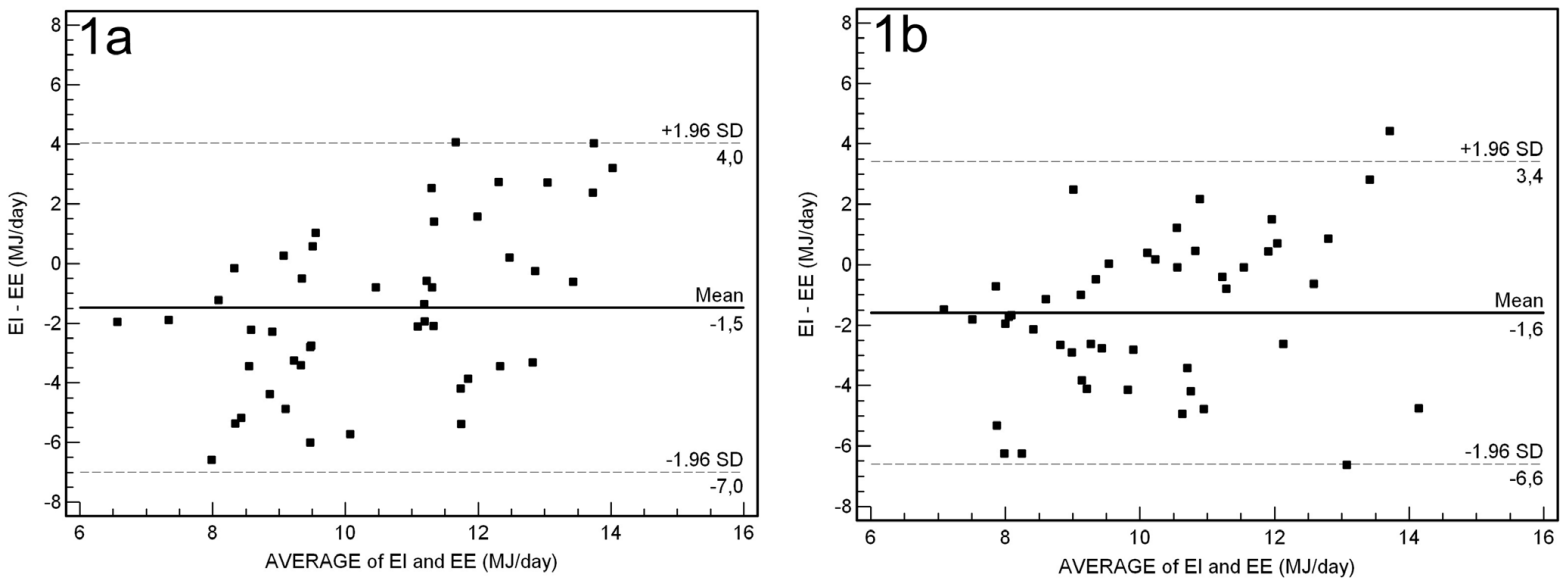
Bland - Altman plots: the baseline difference (Fig.1a) and post-test difference (Fig.1b) between estimated energy expenditure (EE) and estimated energy intake (EI) plotted against the mean of EE and EI. The solid line represents the mean, and the dotted line represents the limits of agreement (plus or minus 1.96 SD).

**Figure 2:**
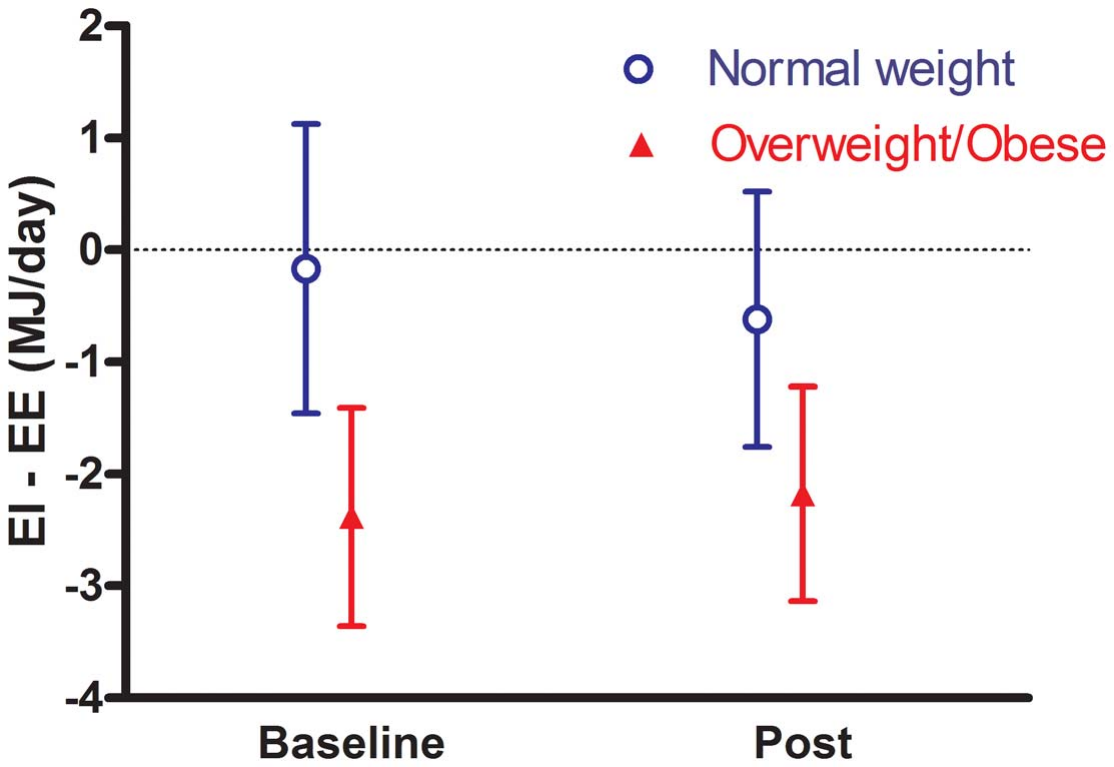
Error bars illustrating mean difference (95% CI) between energy intake (EI) and energy expenditure (EE) in normal weight and overweight/obese participants.

The data were normally distributed and parametric statistical analysis was used to detect differences between EE (SWA) and EI (PFD). Table 1 presents physical characteristics of the participants as means and standard deviations. The accuracy of the reported EI was calculated from the ration EI/EE, for which a value of 1 refers to complete agreement between EI and EE. However, energy intake and energy expenditure may vary largely from day to day and exact agreement between EI and EE over several days in one individual is unlikely. Therefore, the accuracy of the reported EI was assessed partly based on the 95% confidence limits of agreement between EI and EE measured by the DLW method as proposed by Black [29]. Under-reporters were defined as EI/EE<0.80, acceptable reporters were defined as having a ration EI/EE in the range 0.80–1.20, while over-reporters were defined as EI/EE>1.20. Visual agreement between the methods was analysed using the procedure proposed by Bland and Altman [30], using a plot of the difference between the two methods against the average of the measurements (Figure 1a and 1b). This type of plot shows the magnitude of disagreement, spot outliers and any trend. A two-way mixed, single measure, parametric intraclass correlation (ICC) was performed for evaluating the extent of agreement between the SWA and the PFD. Difference in self-reported EI and EE among normal weight and overweight/obese participants were analysed using a paired sample t-test (Table 2). Figure 2 shows error bars illustrating mean difference between EI and EE among normal weight and overweight/obese participants, respectively. A dependent sample t-test was used to analyse whether misreporting of energy intake varied between normal weight and overweight/obese participants. Results were considered statistical significant at p<0.05. Data were analysed using SPSS for Windows release 19.0 (SPSS Inc., Chicago, IL, USA).

**Table 1:** Physical characteristics of the participants (n = 47), energy expenditure (EE) measured with SenseWear Pro_3_ Armband and energy intake (EI) from the pre-coded food diary.

	Baseline		Post-test	
	Mean	SD	Mean	SD
BMI (kg/m^2^)	26.2	3.4	26.5	3.4
Overweight, n (%)	21 (43.8)		22 (45.8)	
Obese, n (%)	8 (16.7)		9 (18.8)	
Percentage fat (%)	26.5	6.6	25.8	6.2
EE (MJ/day)	11.2	1.7	10.9	1.9
EI (MJ/day)	9.7	2.9	9.3	2.5
EI - EE	−1.5	2.8	−1.6	2.5
EI/EE	0.9	0.2	0.9	0.2
Acceptable reporters, n (%)	19 (40.4)		23 (48.9)	
Under-reporters, n (%)	22 (46.8)		21 (44.7)	
Over-reporters, n (%)	6 (12.8)		3 (6.4)	

**Table 2:** Self-reported energy intake (EI) and measured energy expenditure (EE) among normal weight and overweight/obese participants.

	n	EI (MJ/day)	EE (MJ/day)	p-value*
**Baseline**				
Normal weight	18	10.8 (9.3, 12.3)	11.0 (10.0, 11.9)	0.784
Overweight/Obese	29	9.0 (8.0, 10.0)	11.4 (10.8, 11.9)	<0.001
**Post-test**				
Normal weight	16	9.7 (8.4, 11.1)	10.4 (9.3, 11.4)	0.526
Overweight/Obese	31	9.1 (8.2, 10.0)	11.3 (10.6, 11.9)	<0.001

*Dependent sample t-test.

## Results

Mean age of the participants was 68.4 (SD 6.3) years. Table 1 shows that 29 (61%) and 31 (65%) of the participants were categorized as overweight or obese at baseline and post-test, respectively. Mean body fat was 27% at baseline and 26% at post-test. The mean weight remained stable during both periods of data collection (<1 kg daily variance).

The average EI was 17% lower than the measured EE at baseline and 18% lower at post-test.

Bland-Altman plots, showing the difference between EI estimated from the PFD and EE measured by the SWA plotted against the mean of the two methods, are presented in Figure 1a (baseline) and 1b (post-test). Mean difference from Bland-Altman plot for EI and EE was -1.5 MJ/day at baseline and −1.6 MJ/day at post-test and the width of 95% limits of agreement varied from −7.0 to 4.0 MJ/day at baseline and from −6.6 to 3.4 MJ/day at post-test, respectively. A total of 22 (47%) and 21 (49%) participants were under-reporting and 6 (13%) and 3 (6%) were over-reporting energy intake at baseline and post-test, respectively.

The ICCs were 0.30 (95% confidence interval (CI): 0.02, 0.54) at baseline (p = 0.018) and 0.34 (95% CI: 0.06, 0.57) at post-test (p = 0.009), giving 30 to 34% of the variance explained by differences among individuals.

Measured energy expenditure was significantly higher than self-reported energy intake among overweight/obese participants at both baseline and post-test (p<0.001) (Table 2). This relationship was not shown among normal weight participants. Figure 2 shows that mean difference between EI and EE was −0.2 MJ/day (95% CI: −1.5, 1.1) in normal weight participants and −2.4 MJ/day (−3.4, −1.4) in overweight/obese participants at baseline. Similar results were shown from post-test as mean difference between EI and EE was −0.6 MJ/day (−1.8, 0.52) in normal weight and −2.2 MJ/day (−3.1, −1.2) in overweight/obese participants. Among those who underreported EI at baseline, 7 (14.9%) were normal weight and 15 were overweight (31.9%). Among those who underreported EI at post-test, 6 (12.8%) were normal weight and 15 (31.9%) were overweight. Thus, underreporting was significantly more prevalent among overweight/obese participants compared to normal weight participants at both baseline and post-test (p<0.001 for both), respectively.

## Discussion

To our knowledge, the PFD used in the present study has never before been used in this age group. The advantage of this method compared with traditional methods like weighed records and dietary history is that it is less time-consuming for the participants and the researchers to conduct. Most of the participants only used approximately 10–15 minutes per day to complete the PFD.

The present study showed that group average of self-reported EI was underreported by 17–18% compared with EE estimated by the SWA. Applying Bland-Altman plots to the energy data showed a mean difference with a large variance and a scattering of the differences which indicated wide discrepancies between the two methods for individual subjects. Although underreporting was most evident, figure 1a and 1b illustrate the problem with both under- and overreporting of energy intake among the participants. The proportion of participants underreporting EI in the present study was somewhat higher than in other studies (13.6–16.2%) targeting similar age groups [31], [32]. Studies among Norwegian children and adolescents that evaluated EI estimated from the same PFD as used in the present study against EE measured with a physical activity monitor (ActiReg), reported corresponding results underreporting ranging from 18% to 34% [12], [33].

Different factors may explain the misreporting of energy intake. On the basis of ICC, the results from both baseline and post-test indicated that between 30–34% of the variance in EE and EI was explained by differences among individuals. The present study showed a significant relation between underreporting of energy intake and BMI; the EI seemed to be more valid in normal weight participants compared to overweight/obese participants. Previous studies which have focused on identifying predictors of misreporting energy intake, confirm a positive relationship between overweight/obesity and underreporting of energy intake among elderly [16], [31], [34]. A study among 217 elderly women from Perth, Australia, showed higher odds of underreporting in overweight (OR = 2.98, 95% CI: 1.46, 6.09) and obese participants (OR = 5.84, 95% CI: 2.41, 14.14) compared to the rest of the study sample [34]. Furthermore, a study including 2083 elderly Belgian men and women concluded that BMI seemed to be one of the most important factors explaining misreporting [31]. A cohort study among 309 middle-aged Danish men investigated the degree of misreporting of EI and the association between underreporting and previous and current body size [14]. They found that among the participants currently not obese at the mean age of 49 years, underreporting was more than twice as prevalent among those who had been obese at the mean ages of 20 (44%) compared to those who were not obese at this age (21%) [14].

Within a longitudinal study on aging population in Germany, results among 238 female and 105 male participants showed that underreporters (7.6% of females and 16.2% of males), had lower educational level, significantly greater BMI and fat mass compared to adequate reporters [32].

The PFD used in the present study has previously been used in a study among 9 year old participants, and in this age group there was no significant differences in BMI between under-reporters and acceptable reporters (p = 0.77) [33]. In one of two studies among 13 year old girls; however, there was a significant negative relationship between BMI and the difference between EE and EI (EE-EI) (p = 0.003) [12], which is in contrast to most observations [10], [35], [36].

As the volunteers who participated in the present study were a small group of healthy non-smoking men who did not use medication or supplements, they are most properly not representative for the general elderly population. Another limitation is the choice of reference method in the present study. Validation studies of SWA indicate that it underestimates EE compared to doubly labelled water (4.7–12.5%) [19], [20]. Due to this underestimation, even larger underreporting from the recorded EI than observed may have occurred. However, SWA is a less expensive and complicated method compared with the other objective methods, as doubly labeled water and indirect calorimetry. Moreover, studies have concluded that SWA perform similar to or more accurate than other commonly used portable physical activity monitors [33], [37].

It is possible that the participants did change their eating- and physical activity pattern due to increased awareness during the period of diet registration and use of SWA. However, the participants were instructed to maintain their usual daily routines of activity and eating pattern. Finally, the conclusions that have been drawn from the present study are strengthened as similar results were shown at baseline and post-test, respectively.

## Conclusion

In summary, the results indicate that the PFD could be a useful tool for estimating energy intake in normal weight elderly men. As overweight/obese participants underestimated energy intake substantially, the PFD seemed to be less suitable for estimating energy intake in this subgroup of elderly men.
